# Synthesis of Poly(Dimethylmalic Acid) Homo- and Copolymers to Produce Biodegradable Nanoparticles for Drug Delivery: Cell Uptake and Biocompatibility Evaluation in Human Heparg Hepatoma Cells

**DOI:** 10.3390/polym12081705

**Published:** 2020-07-29

**Authors:** Ali Khalil, Saad Saba, Catherine Ribault, Manuel Vlach, Pascal Loyer, Olivier Coulembier, Sandrine Cammas-Marion

**Affiliations:** 1 Laboratory of Polymeric and Composite Materials, Center of Innovation and Research in Materials and Polymers (CIRMAP), University of Mons (UMons), Place du Parc 23, 7000 Mons, Belgium; alikhalil92@hotmail.com; 2 ENSCR, CNRS, ISCR (Institut des Sciences Chimiques de Rennes) - UMR 6226, Univ Rennes, 35000 Rennes, France; 3 INSERM, INRAE, Univ Rennes, Institut NUMECAN (Nutrition Metabolisms and Cancer) UMR_A 1341, UMR_S 1241, F-35000 Rennes, France; saad.saba@univ-rennes1.fr (S.S.); catherine.ribault@univ-rennes1.fr (C.R.); manuel.vlach@univ-rennes1.fr (M.V.)

**Keywords:** poly(dimethylmalic acid) derivatives, polymeric nanoparticles, HepaRG cells, in vitro cytotoxicity, cellular uptake

## Abstract

Hydrophobic and amphiphilic derivatives of the biocompatible and biodegradable poly(dimethylmalic acid) (PdiMeMLA), varying by the nature of the lateral chains and the length of each block, respectively, have been synthesized by anionic ring-opening polymerization (aROP) of the corresponding monomers using an initiator/base system, which allowed for very good control over the (co)polymers’ characteristics (molar masses, dispersity, nature of end-chains). Hydrophobic and core-shell nanoparticles (NPs) were then prepared by nanoprecipitation of hydrophobic homopolymers and amphiphilic block copolymers, respectively. Negatively charged NPs, showing hydrodynamic diameters (Dh) between 50 and 130 nm and narrow size distributions (0.08 < PDI < 0.22) depending on the (co)polymers nature, were obtained and characterized by dynamic light scattering (DLS), zetametry, and transmission electron microscopy (TEM). Finally, the cytotoxicity and cellular uptake of the obtained NPs were evaluated in vitro using the hepatoma HepaRG cell line. Our results showed that both cytotoxicity and cellular uptake were influenced by the nature of the (co)polymer constituting the NPs.

## 1. Introduction

The field of nanotechnologies has grown exponentially in the past decades with the production of a plethora of nanoobjects for biomedical applications including drug delivery. The rationale for drug delivery using nanoparticles (NPs) is based on the fact that most chemotherapies distribute evenly throughout the body resulting in low plasma drug concentration, limited bioavailability at the site where it is needed, rapid disposal, and side-effects in healthy organs [1]. The design of NPs embedding drugs appears as a suitable strategy to achieve prolonged plasma concentration of therapeutic payloads and a higher bioavailability [2]. In addition, the discovery of the enhanced permeability and retention (EPR) effect [3,4,5], defining the extravasation of nanovectors through the disorganized blood vessels within solid tumors, further supported the principle of drug-loaded NPs in rodent models of cancers, which opened perspectives for improving therapeutic indexes of vectorized chemotherapies [3,4,5].

With the aim of improving cancer diagnostics and therapy, the design of stealth and/or targeted nanovectors has led to the publication of a large body of research’s works that hardly translated to humans so far [6,7,8]. To reach clinical application, nanoparticles (NPs) having a wide range of chemistry and architecture (lipids or polymers nanocarriers, inorganic or viral NPs) have been proposed for cancer therapy and imaging [9,10]. Among all the developed NPs, those formulated from hydrophobic homopolymers and amphiphilic block copolymers present a real interest as a result of the versatility in the structure (homopolymers, block-copolymers, star-shaped (co)polymers, etc.) and chemistry (polyesters, polypeptides, polycyanoacrylates, etc.) of the constituting polymeric materials, which can be adjusted to the selected applications by modifying various parameters, including the monomers features, the polymerization procedure, the nature of end-chain and/or side-chain groups (chemical modifications) [7,11,12,13,14,15].

After systemic injection, many plasma proteins form a protein corona at the surface of NPs in a process named opsonization. The binding of protein involves non-specific interactions with abundant plasma proteins such as albumin, but also the recognition by antibodies and proteins of the complement system [16,17], which is part of the innate immune system enhancing the activity of the mononuclear phagocyte system (MPS) to clear pathogens from the body. In order to minimize the opsonization and the non-specific scavenging by MPS, NPs’ features have been optimized through the modulation of their size, shape [1], surface charge [4] and chemical structure [3,5].

When amphiphilic block copolymers are concerned, poly(ethylene glycol), PEG, often plays the role of hydrophilic segments exposed at the surface of the NPs resulting in the “stealth” behavior of the corresponding PEG-based NPs towards opsonins and extending their systemic lifetime [16,17]. Indeed, the PEG segments associated to water molecules form a hydrating layer [18], which acts as a steric shield to prevent the binding of serum opsonins and delay the uptake by MPS. Furthermore, the PEG corona often increases the hydrodynamic diameter of the NPs thereby decreasing the renal clearance [19]. Although the use of PEG has been approved by the US Food and Drug Administration (FDA) for applications in humans [20], this polymer is very slowly degraded conducting to the production of antibody against PEG, even in the blood of healthy donors, probably as a result of the presence of PEG in commercially available products such as cosmetics [21,22]. The presence of such antibody anti-PEG might thus compromise the efficiency of treatments using PEGylated nanovectors. In addition, the unpredicted clearance times of PEGylated compounds lead to accumulation of high molar mass compounds in the liver [23] with unknown toxicological consequences over a long period of time [24].

Consequently, many studies have been conducted to replace PEG by other hydrophilic blocks such as dextran [25] or poly(malic acid) [26,27,28]. Therefore, the synthesis of well-defined amphiphilic block copolymers constituted only by (bio)degradable and biocompatible polymers is of great interest. In this context, chemical or bio-sourced polyesters have long grasped substantial interest for macromolecular science and medical applications because of their specific characteristics, such as mechanical properties, (bio)degradability and biocompatibility [29]. Chemical polyesters can be synthesized by: (i) polycondensation requiring long reaction times and leading to polyesters with limited molar masses and broad dispersities [30,31], (ii) bacteria fermentation possessing no control on structure and molar masses of the polymers [32], or through (iii) ring-opening polymerization (ROP) of the corresponding cyclic esters [33]. Among those cyclic esters, β-lactones, four-membered ring cyclic esters, have a high ring strain easing their ROP, thus offering the possibilities to obtain a wide variety of structures in terms of nature of repeating units (homo and copolymers), end-groups, and molar masses (adjusted by the ratio monomer/initiator) [33,34].

**Scheme 1:**
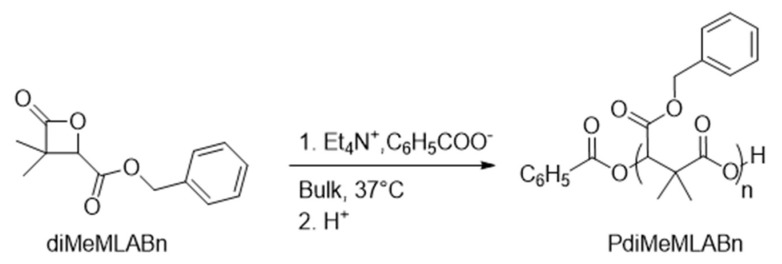
aROP of diMeMLABn in presence of tetraethylammonium benzoate as initiator.

Known for its biocompatibility, dimethylmalic acid can be used to prepare vast range of α,α,β-substituted-β-lactones, whose ROP reactions allow to prepare well-defined biodegradable polymers. However, only few reports have been published on such topic, mainly by Barbaud and collaborators who used tetraethylammonium benzoate to initiate the anionic ROP (aROP) of different α,α,β-substituted-β-lactones (Scheme 1) [35,36,37,38,39,40].

Despites the control over dispersities, molar masses, and structures of the resulting polymers, the major drawback in the use of such initiator for the aROP of the above cited monomers is its potential toxicity towards human cells [41].

In this context, the objectives of the present work were to: (i) synthesize two α,α,β-substituted-β-lactones with different dandling functions (benzyl and hexyl); (ii) set-up initiator systems that initiate aROP of these monomers to obtain well-defined hydrophobic homopolymers and amphiphilic block copolymers; (iii) formulate and characterize the different types of polymeric NPs obtained from either hydrophobic homopolymers or amphiphilic block copolymers; and (iv) evaluate in vitro cytotoxicity and cellular uptake of these NPs using the human HepaRG hepatoma cell line.

## 2. Materials and Methods

### 2.1. Materials and Apparatus

After purification, diMeMLABn and diMeMLAHe lactones were stored at −35 °C. 1-Pyreneacetic acid (PyCOOH, 98%, Aldrich, St. Louis, MO, USA) was dried at 80 °C overnight under vacuum. Tetrahydrofuran (THF, Labscan, 99%) was dried using an MBraun solvent purification system under N_2_. 1-Tert-butyl-4,4,4-tris(dimethylamino)-2,2-bis[tris(dimethylamino) phosphoranyl-idenamino]-2Λ^5^,4Λ^5^-catenadi (phosphazene) (P_4_-*t*-Bu, Sigma, 1 M in hexane under argon) was dried by hexane solvent evaporation. All chemicals were stored and used in a glovebox (<3 ppm O_2_, <1 ppm H_2_O).

Infrared spectroscopy: Fourier transform infra-red (FT-IR) measurements were performed using a Bruker Tensor 27 FT-IR spectrometer using a spectral width ranging from 600 to 4000 cm^−1^, a resolution of 4 cm^−1^ and an accumulation of 32 scans.

Nuclear magnetic resonance spectroscopy: ^1^H-NMR (500 MHz) spectra were recorded using a Bruker AMX-500 apparatus at room temperature and were referenced internally relative to SiMe_4_ (δ 0 ppm) using the residual solvent resonances.

Size exclusion chromatography: Size exclusion chromatography (SEC) was performed in a THF/NEt_3_ (2w% of triethylamine, NEt_3_) mixture at 35 °C using a Triple Detection Polymer Laboratories liquid chromatograph equipped with a refractive index (ERMA 7517), a capillary viscometry, a light scattering RALS (Viscotek T-60) (Polymer Laboratories GPC-RI/CV/RALS) and an automatic injector (Polymer Laboratories GPC-RI/UV) and four columns: a PL gel 10 µm guard column and three PL gel Mixed-B 10 µm columns (linear columns for separation of molar masses ranging from 500 to 10^6^ g/mol).

Dynamic light scattering: Dynamic light scattering (DLS) and zetametry measurements are performed on a Nano-sizer ZS90 (Malvern) at 25 °C, with a He–Ne laser at 633 nm and a detection angle of 90 °C.

Transmission electron microscopy: Transmission electron microscopy (TEM) images were recorded using a Jeol 2100 microscope equipped with a Glatan Orius 200D camera using a 200 KeV accelerating voltage on the THEMIS platform (ISCR – Rennes). Each sample was deposited on a Formvar-carbon film coated on a 300-mesh copper grid. After 6 min, the excess of sample was removed, and a staining was realized with phosphotungstic acid (1 v%).

### 2.2. Synthesis of Monomers and (co)Polymers

**Scheme 2:**
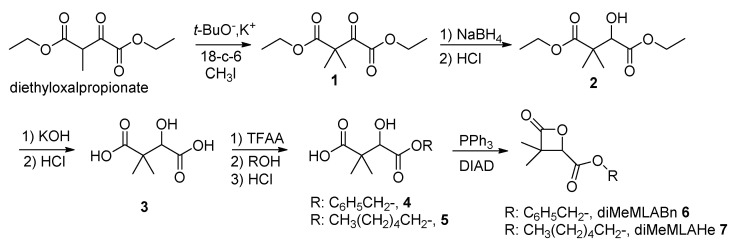
Synthetic route to benzyl and hexyl dimethylmalolactonates.

Synthesis of diMeMLABn and diMeMLAHe: The selected lactones, diMeMLABn and diMeMLAHe, were synthesized from diethyloxal propionate (Scheme 2) as previously described [35,36,37,38,39,40], and characterized by FT-IR and ^1^H-NMR.

*diMeMLABn **6***:

FT-IR: 1750 cm^−1^, ν_CO_ ester; 1850 cm^−1^, ν_CO_ lactone.

^1^H-NMR (500 MHz, CD_3_COCD_3_, δ ppm): 1.12 (s, 3H, CH_3_), 1.50 (s, 3H, CH_3_), 4.90 (s, 1H, CH), 5.25 (s, 2H, CH_2_), 7.30–7.50 (m, 5H, C_6_H_5_).

*diMeMLAHe **7***:

FT-IR: 1750 cm^−1^, ν_CO_ ester; 1850 cm^−1^, ν_CO_ lactone.

^1^H-NMR (500 MHz, CDCl_3_, δ ppm): 0.80 (t, 3H, CH_3_), 1.25–1.40 (m, 9H, CH_3_ + 3*CH_2_), 1.50 (s, 3H, CH_3_), 4.20–4.25 (t, 2H, CH_2_), 4.13 (s, 1H, CH).

Synthesis of PdiMeMLABn_30_: In glove box, 1-pyreneacetic acid (2.6 mg, 0.011 mmol, 1 equiv.) and P_4_-*t*-Bu (2.1 mg, 0.008mmol, ≈ 1equiv.) were introduced in a vial and dissolved in THF (0.5 mL). In another vial, 82 mg of diMeMLABe (0.352 mmol, 32 equiv.) were dissolved in 1 mL of dry THF. The two solutions were mixed ([diMeMLABe]_0_=0.16 M) at room temperature for an appropriate reaction time. The resulting mixture was concentrated to dryness under vacuum. The PdiMeMLABn_30_ homopolymer was precipitated in cold ethanol. The homopolymer samples were characterized (^1^H-NMR and SEC), and kept under inert atmosphere at 4 °C.

^1^H-NMR (500 MHz, CD_3_COCD_3_, δ ppm): 1.15 (m, 3nH, CH_3_), 1.23 (m, 3nH, CH_3_), 4.5 (s, 2H, Pyrene–CH_2_), 5.14 (m, 2nH, CH_2_), 5.3 (m, 1nH, CH), 7.35 (m, 5nH, C_6_H_5_), 7.98–8.34 (m, 9H, pyrene); M_NMR_ = 7000 g/mol, n = 30.

SEC (THF/NEt_3_, 1 mL/min): Mn = 2700 g/mol; Đ = 1.20.

Synthesis of PdiMeMLAHe_30_: In glove box, 1-pyreneacetic acid (1.8 mg, 0.0069 mmol, 1 equiv.) and P_4_-*t*-Bu (2.1 mg, 0.0068 mmol, ≈1 equiv.) were introduced in a vial and dissolved in THF (1 mL). In another vial, 52 mg of diMeMLAHe (0.228 mmol, 33 equiv.) were dissolved in 2 mL of dry THF. The two solutions were mixed ([diMeMLAHe]_0_=0.15 M) at room temperature for an appropriate reaction time. The resulting mixture was concentrated to dryness under vacuum. The recovered PdiMeMLAHe_30_ homopolymer was then precipitated in cold heptane. The homopolymer samples were characterized (^1^H-NMR and SEC), and kept under inert atmosphere at 4 °C.

^1^H-NMR (500 MHz, CD_3_COCD_3_, δ ppm): 0.91 (m, 3nH, CH_3_–CH_2_), 1.34 (m, 9nH, CH_3_, 3*CH_2_), 1.39 (m, 3nH, CH_3_), 1.66 (m, 2nH, CH_2_), 4.17 (m, 2nH, CH_2_–O), 4.5 (s, 2H, Pyrene–CH_2_), 5.34 (m, 1nH, CH), 8.03–8.42 (m, 9H, pyrene); M_NMR_ = 6800 g/mol, n = 30.

SEC (THF/NEt_3_, 1 mL/min): Mn = 5500 g/mol; Đ = 1.16.

Synthesis of amphiphilic block copolymers: In glove box, 1-pyreneacetic acid (1.8 mg, 0.0069 mmol, 1 equiv.) and P_4_-*t*-Bu (4.3 mg, 0.0068 mmol, ≈ 1equiv.) were introduced in a vial and dissolved in THF (0.5 mL). In another vial, 50 mg of diMeMLAHe (0.226 mmol, 32 equiv., for the copolymer PdiMeMLABn_54_-*b*-PdiMeMLAHe_29_) or 77 mg of diMeMLAHe (0.339 mmol, 49 equiv., for the copolymer PdiMeMLABn_27_-*b*-PdiMeMLAHe_50_) were dissolved in 1 mL of dry THF. The two solutions were mixed ([diMeMLAHe]_0_=0.16 M) at room temperature for an appropriate reaction time. After full conversion (determined by FTIR), a solution of 80 mg of diMeMLABn (0.345 mmol, 49 equiv., for the copolymer PdiMeMLABn_54_-*b*-PdiMeMLAHe_29_) in 0.8 mL THF or a solution of 39 mg of diMeMLABn (0.166 mmol, 24 equiv., for the copolymer PdiMeMLABn_27_-*b*-PdiMeMLAHe_50_) in 0.39 mL of THF was added to the reaction mixture ([diMeMLABn]=0.16 M). After full conversion, the resulting mixture was concentrated to dryness under vacuum. The recovered PdiMeMLABn_n_-*b*-PdiMeMLAHe_m_ copolymer was dissolved in a minimum amount of acetone and then precipitated in cold ethanol. The copolymer samples were characterized (^1^H-NMR and SEC), and kept under inert atmosphere at 4 °C.

^1^H-NMR (500 MHz, CD_3_COCD_3_, δ ppm, identical for both block copolymers excepted the relative integration values): 0.89 (m, 3H, CH_3_–CH_2_), 1.30 (m, 18H, 4*CH_3_, 3*CH_2_), 1.65 (m, 2H, –CH_2_–CH_2_–O), 4.14 (m, 2H, –CH_2_–CH_2_–O), 4.55 (s, 2H, Pyrene–CH_2_), 5.12 (m, 2H, C_6_H_5_–CH_2_–O), 5.29 (m, 2H, 2*CH), 7.34 (m, 5H, C_6_H_5_), 8.04–8.41 (m, 9H, pyrene);

M_NMRPdiMeMLABn_ = 12,600 g/mol (n = 54), M_NMRPdiMeMLAHe_ = 6600 g/mol (m = 29)

M_NMRPdiMeMLABn_ = 6400 g/mol (n = 27), M_NMRPdiMeMLAHe_ = 11,800 g/mol (m = 50)

SEC (THF/NEt_3_, 1 mL/min)

PdiMeMLABn_54_-*b*-PdiMeMLAHe_29_: Mn = 12,000 g/mol; Đ = 1.25.

PdiMeMLABn_27_-*b*-PdiMeMLAHe_50_: Mn = 7400 g/mol; Đ = 1.31.

Both hydrophobic copolymers were then subjected to catalytic hydrogenolysis allowing the specific deprotection of the carboxylic acid lateral groups. They were dissolved in a THF/Ethanol (50/50 v/v%) mixture and 20 wt% of Pd/C (relative amount to benzyl groups) were added. The mixtures were placed under atmospheric pressure of H_2_ and left under vigorous stirring overnight. The black suspensions were filtered over celite to eliminate the Pd/C and solvents were evaporated under vacuum thus leading to the expected amphiphilic block copolymers characterized by ^1^H-NMR.

^1^H-NMR (500 MHz, CD_3_COCD_3_, δ ppm, identical for both block copolymers excepted the relative integration values): 0.89 (m, 3H, CH_3_–CH_2_), 1.30 (m, 18H, 4*CH_3_, 3*CH_2_), 1.65 (m, 2H, –CH_2_–CH_2_–O), 4.14 (m, 2H, –CH_2_–CH_2_–O), 4.55 (s, 2H, Pyrene–CH_2_), 5.29 (m, 2H, 2*CH), 8.04–8.41 (m, 9H, pyrene);

M_NMRPdiMeMLA_ = 7720 g/mol (n = 54), 50%; M_NMRPdiMeMLAHe_ = 6600 g/mol (m = 29), 50%.

M_NMRPdiMeMLA_ = 3850 g/mol (n = 27), 25%; M_NMRPdiMeMLAHe_ = 11,800 g/mol (m = 50), 75%.

### 2.3. Formulation of Nanoparticles

Empty nanoparticle formulations based on hydrophobic homopolymers: In an Eppendorf, 2.5 mg of homopolymer were dissolved in 150 µL DMF and added dropwise to 2 mL of distilled water under 1400 rpm stirring speed. The solution was stirred for 20 min. The solution was then passed through a G25 Sephadex column leading to 3.5 mL NP suspensions which were characterized by DLS, zetametry and TEM.

NPs PdiMeMLABn_30_: M = 7000 g/mol; Final concentration = 0.714 mg/mL; Dh = 110 nm; PDI = 0.15; Zeta potential = −34 mV.

NPs PdiMeMLAHe_30_: M = 6800 g/mol; Final concentration = 0.714 mg/mL; Dh = 140 nm; PDI = 0.07; Zeta potential = −47 mV.

Empty nanoparticle formulations based on amphiphilic block copolymers: In a vial, 10 mg of PdiMeMLA_54_-*b*-PdiMeMLAHe_29_ or 5 mg of PdiMeMLA_27_-*b*-PdiMeMLAHe_50_ were solubilized in 1 mL of acetone then added dropwise to 2 mL of aqueous phase under 1400 rpm stirring speed. The solution was stirred for 20 min and acetone was removed under reduced pressure. The final volumes of the NPs’ suspensions were adjusted to 2 mL (if necessary) by addition of the needed volume of aqueous phase.

NPs PdiMeMLA_54_-*b*-PdiMeMLAHe_29_: M = 14,600 g/mol; Final concentration = 5 mg/mL; Aqueous phase: water; Dh = 50 nm; PDI = 0.08; Zeta potential = −36 mV.

NPs PdiMeMLA_27_-*b*-PdiMeMLAHe_50_: M = 15,500 g/mol; Final concentration = 2.5 mg/mL; Aqueous phase: PBS; Dh = 110 nm; PDI = 0.20; Zeta potential = −32 mV.

DiR loaded nanoparticle formulations based on hydrophobic homopolymers: In an Eppendorf, 2.5 mg of homopolymer were solubilized in 135.5 µL of DMF and 14.5 µL of a DiR/DMF solution at a concentration of 1 mg/mL (0.5 wt% of the mass of the polymer) were added. This mixture was then added dropwise to 2 mL of distilled water under 1400 rpm stirring speed. The solution was stirred for 20 min. The solution was then passed through a G25 Sephadex column leading to 3.5 mL of DiR loaded NPs’ suspensions which were characterized by DLS and zetametry.

NPs PdiMeMLABn_30_[DiR]: M = 7000 g/mol; Final concentration = 0.714 mg/mL; Dh = 130 nm; PDI = 0.11; Zeta potential = −37 mV.

NPs PdiMeMLAHe_30_[DiR]: M = 6800 g/mol; Final concentration = 0.714 mg/mL; Dh = 110 nm; PDI = 0.12; Zeta potential = −53 mV.

DiR loaded nanoparticle formulations based on amphiphilic block copolymers: In a vial, 10 mg PdiMeMLA_54_-*b*-PdiMeMLAHe_29_ or 5 mg of PdiMeMLA_27_-*b*-PdiMeMLAHe_50_ were solubilized in a total volume of 1 mL of acetone: 900 µL of acetone and 100 µL of a DiR/acetone at 1 mg/mL (1 wt% of the mass of the block copolymer) for the PdiMeMLA_54_-*b*-PdiMeMLAHe_29_ block copolymer; 950 µL of acetone and 50 µL of a DiR/acetone at 1 mg/mL (1 wt% of the mass of the block copolymer) for the PdiMeMLA_27_-*b*-PdiMeMLAHe_50_ block copolymer. These solutions were then added dropwise to 2 mL of aqueous phase under 1400 rpm stirring speed. The solution was stirred for 20 min and acetone was removed under reduced pressure. The samples were then passed through a G25 Sephadex column leading to 3.5 mL of DiR loaded NPs’ suspensions which were characterized by DLS and zetametry.

NPs PdiMeMLA_54_-*b*-PdiMeMLAHe_29_[DiR]: M = 14,600 g/mol; Final concentration = 2.857 mg/mL; Aqueous phase: water; Dh = 60 nm; PDI = 0.09; Zeta potential = −30 mV.

NPs PdiMeMLA_27_-*b*-PdiMeMLAHe_50_[DiR]: M = 15,500 g/mol; Final concentration = 1.429 mg/mL; Aqueous phase: PBS; Dh = 130 nm; PDI = 0.18; Zeta potential = −48 mV.

### 2.4. In Vitro Assays

HepaRG culture: For all the studies, proliferating progenitor HepaRG cells were seeded at a density of 3.10^4^ cells/cm^2^ and cultured as previously described [42,43,44], in William’s E medium (Lonza, Basel, Switzerland) supplemented with 50 units/mL penicillin and 50 µg/mL streptomycin, 2 mM of glutamine (Gibco), 5 mg/L of insulin (Sigma), 10^−5^ mM hydrocortisone hemisuccinate and 10% of fetal calf serum (Lonza). Two weeks after plating, the cells were maintained for two more weeks in the same William’s E medium further supplemented with 2% dimethylsulfoxide (DMSO) in order to obtain the full hepatocyte differentiation [42,43,44].

In vitro DiR loaded NPs uptake by HepaRG cells: For the cell uptake assay of NPs, HepaRG cells were plated in 24 well plates and cultured as mentioned above. The culture medium of HepaRG cells was renewed and PdiMeMLABn-based NPs loaded with DiR were added to the wells for 24 h. After incubation, culture media were discarded, the cell monolayers were washed once with PBS then detached with trypsin and analyzed by flow cytometry using a Becton Dickinson le LSRFortessa™ X-20 (cytometry core facility of the Biology and Health Federative research structure Biosit, Rennes, France). Dot plots of forward scatter (FSC: x axis, size of events), the side scatter (SSC: y axis, structure of events) allowed to gate the viable cells prior detecting the fluorescence emitted by DiR-loaded NPs using the BV786-1A channel. Two parameters were analyzed: the percentage of positive cells that internalized NPs and the intensity of fluorescence (mean of fluorescence) reflecting the accumulation of fluorescent NPs within the cells.

In vitro NPs cytotoxicity assays using HepaRG cells: Cell viability was assessed by measuring the relative intracellular ATP content using CellTiter-Glo Luminescent Cell viability assay (Promega, Madison, WI, USA). After treatment, cells were incubated with the CellTiter-Glo reagent for 10 min. Lysate was transferred to an opaque mutli-well plate and luminescent signal was quantified at 540 nm using the Polarstar Omega microplate reader (BMG Labtech, Ortenberg, Germany). Cell viabilities in treated cells were expressed as the percentage of the luminescence values obtained in untreated cells, which were arbitrary set as 100%.

Statistical analyses: Statistical analyses were performed using a one-way ANOVA followed by the Kruskal–Wallis post-test or Dunn’s multiple comparison tests. Statistically significant variations after treatment were compared with controls using Student’s t test with Excel software. * *p* < 0.05; ** *p* < 0.01.

## 3. Results and Discussion

The general objective of this work was to prepare biocompatible and (bio)degradable macromolecular systems prepared from (co)polymers whose repeating units derive from dimethylmalic acid, a biocompatible and low molar mass molecule, by anionic ring-opening polymerization (aROP) of α,α,β-trisubstituted β-lactones. Indeed, it was shown that the presence of two methyl groups on the ring of these monomers leads to a better control of the polymerization reaction by avoiding back side attack transfer reaction (α deprotonation in anionic propagation reaction) on the polymer chain [35,36,37,38,39,40].

### 3.1. Synthesis and Characterization of diMeMLABn and diMeMLAHe

The selected α,α,β-trisubstituted β-lactones were synthesized starting from diethyloxalpropionate, following a procedure initially described by Barbaud et al. (Scheme 2) [35].

The benzyl dimethylmalolactonate (diMeMLABn, 6) was synthesized to lead either to a hydrophobic homopolymer or, after catalytic hydrogenolysis, to the hydrophilic block of the amphiphilic block copolymer, while the hexyl dimethylmalolactonate (diMeMLAHe, 7) was synthesized to constitute both the hydrophobic homopolymer and the hydrophobic block of the amphiphilic block copolymer. As shown in Scheme 2, diethyloxalpropionate was transformed to diethyl-3,3-dimethyl-2-ketosuccinate 1 in presence of potassium *tert*-butoxide, methyl iodide and 18-crown-6 ether (18-c-6). The ketosuccinate 1 was reacted with sodium borohydride to lead to diethyl-3,3-dimethylmalate 2 whose ester functional groups were hydrolyzed under basic conditions to lead to 3,3-dimethylmalic acid 3. The diacid 3 was then treated with trifluoroacetic anhydride (TFAA) followed by the addition of the selected alcohol, benzyl alcohol or 1-hexyl alcohol, leading to the corresponding monoesters 4 and 5 which were transformed into the expected diMeMLABn 6 and diMeMLAHe 7 by Mistunobu reaction [33,34]. After purifications, the two synthesized lactones showed ^1^H-NMR spectra in agreement with those given in the literature [35,36].

### 3.2. Synthesis and Characterization of Hydrophobic Homopolymers and Amphiphilic Block Copolymers Based on Pdimemla Derivatives

**Scheme 3:**
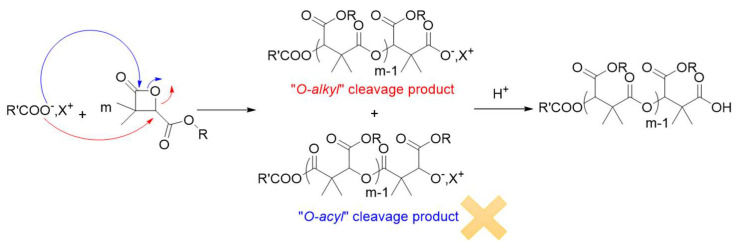
aROP of α,α,β-trisubstituted β-lactones in presence of a carboxylated initiator.

The next step of the present work was to prepare well-defined and well-characterized dimethylmalic acid-based homo- and block copolymers for applications as drug delivery systems. Therefore, the polymerization procedure has to be controlled (molar masses and polymers structures) with no-side reactions, and anionic polymerization is a perfect tool to polymerize β-lactones in a controlled way [45]. Such monomers are generally polymerized by aROP in presence a carboxylated initiator (RCOO^−^ X^+^), instead of an alkoxide initiator, via an “O-alkyl” bound cleavage of the monomer, leading quantitatively to carboxylic acid end-capped (co)polymers (Scheme 3).

Since the (co)polymers prepared may be used for biomedical application, the biocompatibility of the resulting products is of prime importance. To that end, carboxylate initiators were prepared from carboxylic acid deprotonated by phosphazene bases. The reason behind choosing phosphazene bases as deprotonating agents of carboxylic acid is their high basic character (high pK_b_ values). Indeed, phosphazene bases are organobases that contain a central phosphorous atom [P(V)] bonded to four nitrogen functions of three amine and one imine substituents. They are classified as P_n_ bases, based on the number (n) of phosphorous atoms in the molecule. Their basicity is reflected by the number of the triaminoiminophosphorane units: P_4_ bases, the strongest of the phosphazene bases, show basicity comparable to organolithium compounds (^MeCN^pK_BH+_ of P_4_-*t*-Bu, = 41.9, ^MeCN^pK_BH+_ of P_1_-*t*-Bu = 26.9) [46,47,48,49,50]. Beside the choice of the deprotonating agent, the selection of the carboxylic acid (initiator) is also crucial, and 1-pyreneacetic acid has been selected as a non-halogenated carboxylic acid. By comparing the efficiency of the couples 1-pyreneacetic acid/P_1_-*t*-Bu and 1-pyreneacetic acid/P_4_-*t*-Bu for the aROP of diMeMLABn in terms of polymerization rate and control, P_4_-*t*-Bu phosphazene base has been chosen as deprotonating agent because it leads to 100% monomer conversion in few hours with a good control over the molar masses and polymer structure, while the couple 1-pyreneacetic acid/P_1_-*t*-Bu allowed the conversion of only 50% of diMeMLABn in 2.5 days [48]. Assuming that the pyrenecarboxylate moieties initiated the polymerization reaction, experimental molar masses can be calculated by ^1^H-NMR spectroscopy from the relative intensity of methine group of the diMeMLABn monomer repeating units at δ = 5.3 ppm (peak “b”) and proton signals of the initiating pyrene ring (peaks “f”) in the range of δ = 7.9–8.3 ppm (Supplementary Figure S1).

In this context, diMeMLABn and diMeMLAHe were thus polymerized using this 1-pyreneacetic acid/P_4_-*t*-Bu initiator/base couple, with a ratio monomer/initiator selected to obtain homopolymers with a theoretical molar mass of 7500 g/mol (Table 1).

The ^1^H-NMR spectra and SEC chromatograms of the purified homopolymers agreed with both the homopolymer structures and molar masses. The molar masses calculated from the ^1^H-NMR spectra (Table 2) are closed to the theoretical ones while the homopolymers’ dispersities (measured by SEC) are low (Đ ≤ 1.20) attesting of the good control of both polymerization reactions. However, it is important to note that despite drastic purification procedures, traces of P_4_-*t*-Bu are present in PdiMeMLABn_30_ and PdiMeMLAHe_30_ samples as highlighted by the presence of peaks corresponding to this phosphazene base in both ^1^H-NMR spectra (around 2.9 mol% of P_4_-*t*-Bu for each homopolymer).

**Scheme 4:**
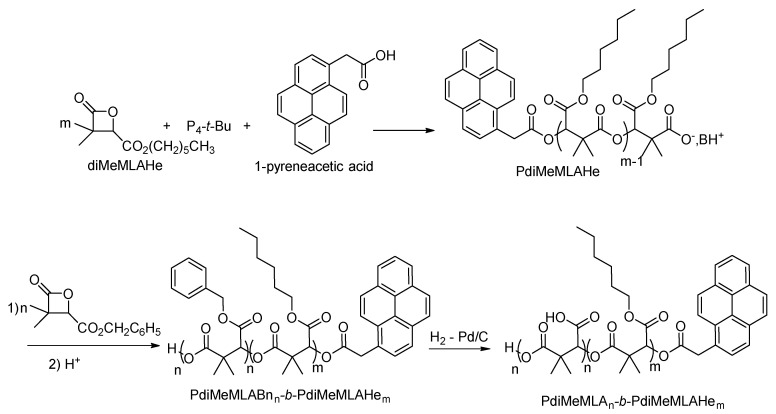
Synthetic procedure leading to amphiphilic block copolymers.

Incorporating two monomers with different physicochemical properties in one copolymer is of great interest, especially for biomedical applications. Copolymerization brings the advantages and properties of both monomers into one macromolecular structure, and several copolymers have already been synthesized and used for biomedical applications [28,36,37,38,51,52,53,54]. Since we have previously shown the effectiveness of the 1-pyreneacetic acid/P_4_-*t*-Bu initiator/base system for the homopolymerization of both diMeMLABn & diMeMLAHe, the same initiator/base system has been selected to polymerize sequentially diMeMLAHe and diMeMLABn in two ratios allowing to modulate the hydrophobic/hydrophilic balance of the final amphiphilic block copolymers (Scheme 4). Indeed, this balance is a key parameter for the preparation of well-defined nanoobjects showing adapted properties for biomedical applications [26,54].

Therefore, diMeMLAHe was polymerized first using 1-pyreneacetic acid/P_4_-*t*-Bu initiator/base system. Once all this lactone was consumed, as attested by the disappearance of the lactonic band at 1850 cm^−1^ on the FT-IR spectrum, the diMeMLABn was added into the reaction mixture in order to build the second block. As shown in Table 2, two diMeMLAHe/diMeMLABn initial ratios were used, thus leading to two block copolymers having similar global molar masses but different lengths of each block.

**Figure 1:**
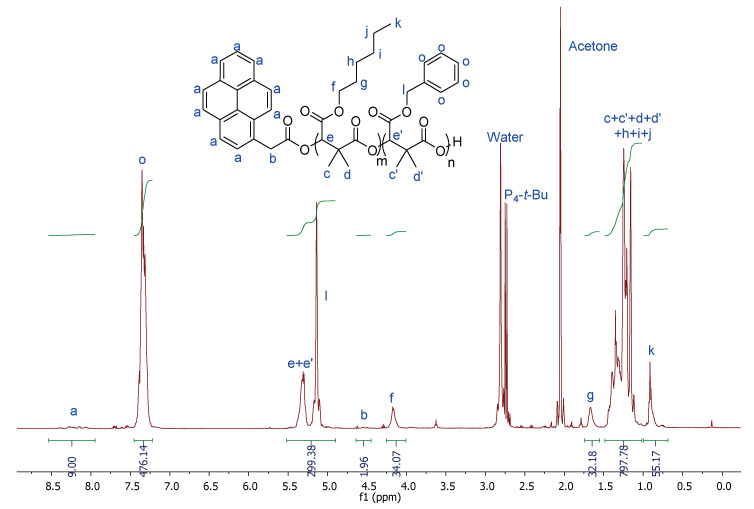
^1^H-NMR (500 MHz, Acetone-d_6_, 23 °C) spectrum of PdiMeMLABn_54_-*b*-PdiMeMLAHe_29_ (Table 2, Entry 1).

In the ^1^H-NMR spectrum of PdiMeMLABn_54_-*b*-PdiMeMLAHe_29_ (Table 2, Entry 1), signals “H_f_” and “H_I_” directly allow determining the composition of each block while their comparison to the signal “H_a_” gives access to the molar mass of each block (Figure 1). As indicated by the results from Table 2, the theoretical molar masses of each block are in good agreement with those calculated from ^1^H-NMR spectra. Moreover, the low dispersity (Đ ≤ 1.31) of both samples demonstrated the good control of these sequential aROPs and the effective synthesis of the selected block copolymers with the expected structures. However, as for homopolymers, ^1^H-NMR spectra have highlighted the presence of phosphazene base P_4_-*t*-Bu traces (around 1 mol% in each sample) in the purified block copolymers despite the drastic purification procedures which were applied.

Because of the presence of ester bounds in block copolymers’ back-bone and lateral chains, catalytic hydrogenolysis using palladium on activated charcoal has been selected as a mild and specific deprotection method of benzyl esters [55]. Both hydrophobic block copolymers were thus submitted to catalytic hydrogenolysis in presence of 20wt% of Pd/C under atmospheric pressure of hydrogen, thus leading to the expected amphiphilic block copolymers, PdiMeMLA_54_-*b*-PdiMeMLAHe_29_ and PdiMeMLA_27_-*b*-PdiMeMLAHe_50_ (Table 3).

### 3.3. Formulation and Characterization of Nanoparticles

Towards the end of the 1980s, Kataoka et al. have described the preparation of polymeric NPs from amphiphilic macromolecules for the delivery of bioactive molecules (drugs, fluorescent probes, proteins etc.) [56]. Several more or less complex methods are used to formulate NPs, among which nanoprecipitation of hydrophobic and amphiphilic block copolymers, first described by Fessi et al. [57,58], were used by Barbaud et al. to prepare Warfarin-loaded NPs from random or block copolymers based on PdiMeMLA derivatives [59]. Nanoprecipitation conditions were slightly modified to be adjusted to the physico-chemical properties of hydrophobic homopolymers and amphiphilic block copolymers produced in this work. Indeed, the synthesized homopolymers and amphiphilic block copolymers exhibit much lower global molar masses than the ones used by Barbaud et al. [59]. Hydrophobic homopolymers, dissolved in DMF, were added dropwise into distilled water under vigorous stirring for 20 min. The obtained NPs suspensions were purified by exclusion chromatography through a Sephadex column to obtain NPs suspensions with a final concentration of 0.714 mg/mL, analyzed by DLS and zetametry (Table 4). The PdiMeMLAHe_30_ based NPs have a slightly higher hydrodynamic diameter (Dh) than the one observed for PdiMeMLABn_30_ based NPs (Table 4). Due to the linear and flexible character of the hexyl lateral chains, compaction of the polymeric chains to form the NPs might be lower than the one observed with the benzyl lateral chains of the PdiMeMLABn_30_ based NPs. However, PdiMeMLAHe_30_ are of interest to study the effect of the lateral chains’ nature of both drug encapsulation efficiency and NPs cytotoxicity. Moreover, both batches of NPs suspensions have a low dispersity (PDI < 0.20) demonstrating the good homogeneity of the NPs. On the other hand, the value of zeta potential is an important indication of the stability of the NPs. Indeed, the higher the magnitude of the zeta potential, the more stable the NPs [60].

**Figure 2:**
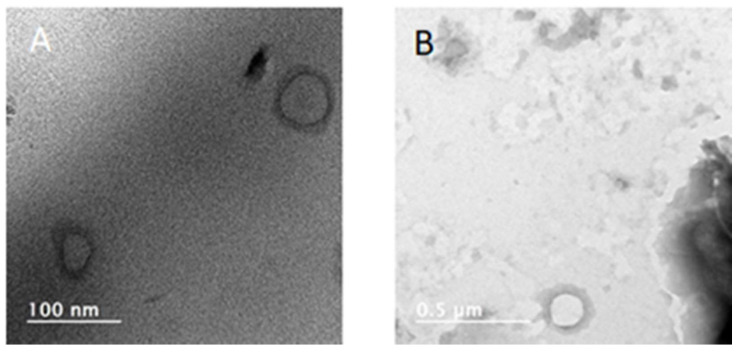
TEM images of (**A**) PdiMeMLABn_30_, and (**B**) PdiMeMLAHe_30_ based NPs.

All the prepared NPs exhibit negative zeta potential ranging from −37 mV to −53 mV highlighting their good stability. The negatively charged surface might be partially generated from carboxylate functional end groups of homopolymers coming from their synthesis via the aROP of the corresponding monomer. Finally, the TEM images confirm both the size of these NPs and the low sample dispersity and that NPs based on PdiMeMLABn_30_ and PdiMeMLAHe_30_ exhibited a spherical shape (Figure 2).

Amphiphilic block copolymers containing hydrophilic and hydrophobic blocks are widely used to prepare NPs having a core-shell structure with the hydrophilic segment constituting a hydrophilic corona, which reduces the recognition by the immune system, whereas the hydrophobic segment drives the formation of NPs through hydrophobic interactions constituting the inner core in which hydrophobic drugs can be encapsulated. Therefore, PdiMeMLA_54_-*b*-PdiMeMLAHe_29_ and PdiMeMLA_27_-*b*-PdiMeMLAHe_50_ have been used to formulate the corresponding core-shell NPs by nanoprecipitation (Table 5), with conditions adjusted to the nature of the considered amphiphilic block copolymers. For PdiMeMLA_27_-*b*-PdiMeMLAHe_50_ block copolymer, well-defined NPs have been obtained when PBS at pH 7.2 was used as aqueous phase. Moreover, we have shown that the block copolymer concentration in the organic phase (5 mg/mL and 10 mg/mL), and consequently in the aqueous phase (2.5 mg/mL and 5 mg/mL), has no influence on both the hydrodynamic diameter (around 110 nm) and dispersity (PDI around 0.20) of the resulting NPs suspensions (data not shown). PdiMeMLA_27_-*b*-PdiMeMLAHe_50_ based NPs were thus prepared at a final block copolymers concentration in PBS of 2.5 mg/mL.

**Figure 3:**
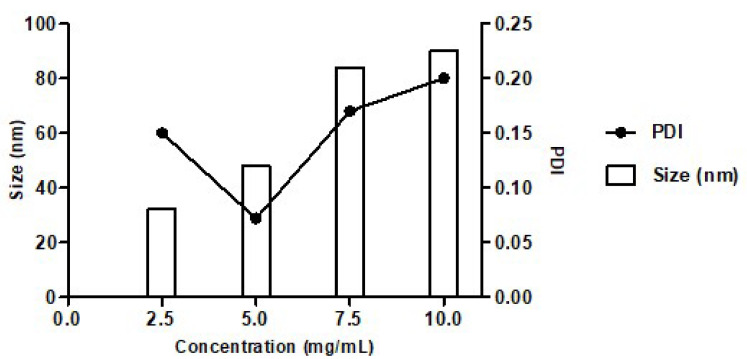
Variation of hydrodynamic diameter and PDI of PdiMeMLA_54_-*b*-PdiMeMLAHe_29_ based NPs with different final concentrations in water.

Conversely, PdiMeMLA_54_-*b*-PdiMeMLAHe_29_ block copolymers lead to well-defined NPs when water was used as non-solvent. We have also studied the influence of initial and final block copolymer concentration on NPs hydrodynamic diameter and dispersity. Four initial concentrations (5, 10, 15, and 20 mg/mL) in acetone have been tested. After evaporation of the acetone under vacuum, the resulting nanoparticle suspensions, with block copolymer concentration of 2.5, 5, 7.5 and 10 mg/mL, were analyzed by DLS (Figure 3). When the concentration increased from 2.5 to 10 mg/mL, the NPs hydrodynamic diameter increased from 30 to 100 nm, while the PDI values first decreased from 0.2 to a minimum inflection point of PDI value of 0.07 and then increased up to 0.2. In aqueous solutions, amphiphilic molecules orientate themselves so that the hydrophobic blocks are removed from the aqueous environment in order to achieve a state of minimum free energy. As the concentration of block copolymer in solution increases, the free energy of the system begins to rise due to unfavorable interactions between water molecules and the hydrophobic region of the amphiphilic block copolymer, resulting in the formation of micelles.

**Figure 4:**
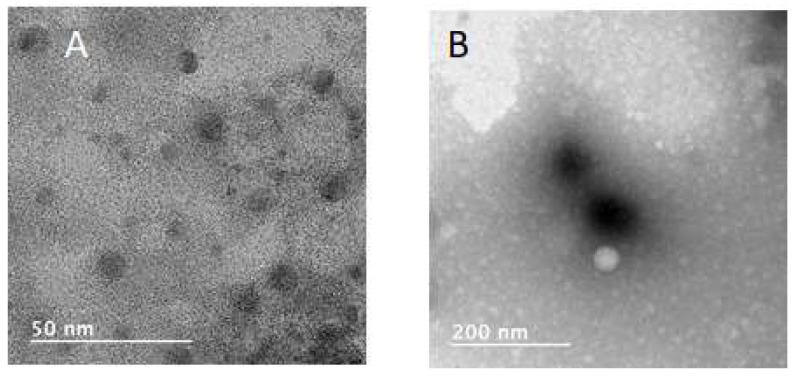
TEM images of (**A**) PdiMeMLA_54_-*b*-PdiMeMLAHe_29_, and (**B**) PdiMeMLA_27_-*b*-PdiMeMLAHe_50_ based NPs.

The same variation in size and PDI values of polymeric NPs upon nanoprecipitation, as shown in Figure 3, was reported for the nanoprecipitation of PCL [61]. Considering that well-defined (lowest PDI) NPs with diameters as small as possible (10–1000 nm) are required for drug delivery application, the batch prepared by nanoprecipitation of PdiMeMLA_54_-*b*-PdiMeMLAHe_29_ at the concentration of 5 mg/mL and producing NPs with a hydrodynamic diameter of ~50 nm and the lowest PDI (~0.07) is the best condition compared to the three others. The negative zeta potential of NPs constituted by PdiMeMLA-based block copolymers are generated by the presence of negatively charged carboxylate anions dandling from the hydrophilic block of the copolymer. These results are comparable with the ones obtained for poly(malic acid)-*b*-poly(3-hydroxybutyrate) (PMLA-*b*-PHB) block copolymer-based NPs for which it was reported a negative values of zeta potential with values varying from −32 mV to −52 mV [53]. As shown by Figure 4, NPs formed by PdiMeMLA-based block copolymers also display quite a round shape with a lower diameter than those observed by DLS. The hydrophilic corona are not visible by TEM because images are taken in a dry state.

With the goal to evaluate the in vitro cell uptake of these NPs, the fluorescence probe 1,1′-dioctadecyl-3,3,3′,3′-tetramethylindotricarbocyanine iodide (DiR) has been embedded into hydrophobic homopolymers and amphiphilic block copolymers based NPs using nanoprecipitation methods established for the formulation of the corresponding empty NPs. Therefore, DiR was added into the organic solvent containing homopolymers or block copolymers and the blue/green DiR/(co)polymers solutions were nanoprecipitated. The blue/green slightly turbid NPs suspensions were passed through a G25 Sephadex column in order to eliminate the unloaded DiR, which is retained on the top of the column. We have shown that nearly all the DiR was encapsulated into the core of NPs since no free DiR was detected on the top of the Sephadex column. DiR-loaded hydrophobic homopolymers and amphiphilic block copolymers NPs were next analyzed by DLS (Table 6). The encapsulation of the fluorescence probe had no influence on both the hydrodynamic diameter and dispersity since both empty and DiR-loaded NPs have similar hydrodynamic diameters and dispersities. Before performing in vitro cell assays, stability of NPs suspensions was also measured to evaluate their stability under storage at 4 °C and after incubation at 37 °C. Only slight fluctuations in the values of hydrodynamic diameter and PDI were recorded highlighting the obvious excellent stability of these NPs under the incubation conditions (data not shown).

### 3.4. In Vitro Cell Viability Assays

The liver is the major site of metabolism of xenobiotics prior to their elimination from the body and, as such, the toxicity of NPs is important to evaluate using hepatic cell models. In order to assess the biocompatibility of the NPs produced in this work, we used the human HepaRG hepatoma cell line. The HepaRG cells are undifferentiated bipotent progenitor cells actively proliferating that have the ability to differentiate into quiescent hepatocyte-like cells [43,44], which express most of the liver specific functions including drug metabolism enzymes [62,63,64,65]. The HepaRG cells are considered to be one of the most relevant hepatic cell models to examine the cytotoxic effect of xenobiotics both in proliferating progenitor and differentiated quiescent cells as well as the cellular uptake of polymeric NPs [53].

**Figure 5:**
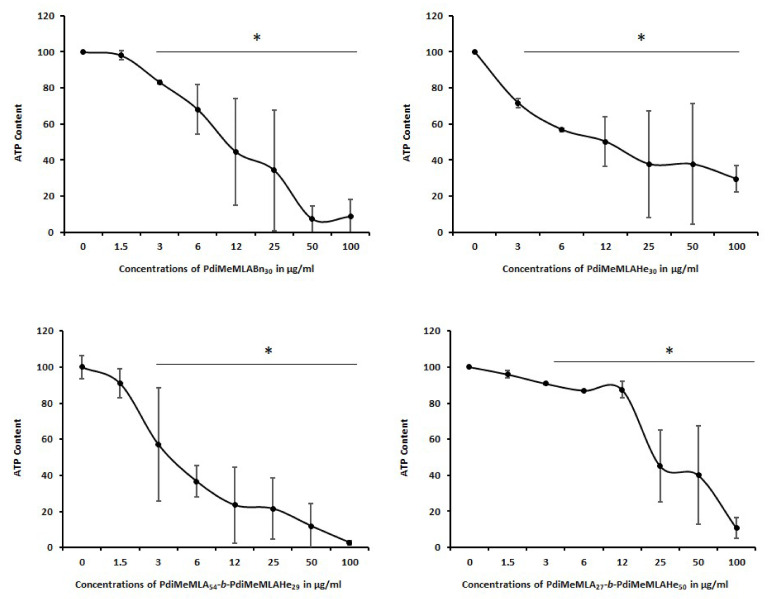
Progenitor HepaRG cell viability evaluated by measuring the ATP content at 24 h of incubation with increasing concentrations of (co)polymers PdiMeMLA_54_-*b*-PdiMeMLAHe_29_, PdiMeMLA_27_-*b*-PdiMeMLAHe_50_, PdiMeMLABn_30_ and PdiMeMLAHe_30_ NPs. Statistics: * *p* < 0.05 for treated cultures versus untreated control cultures (0).

**Figure 6:**
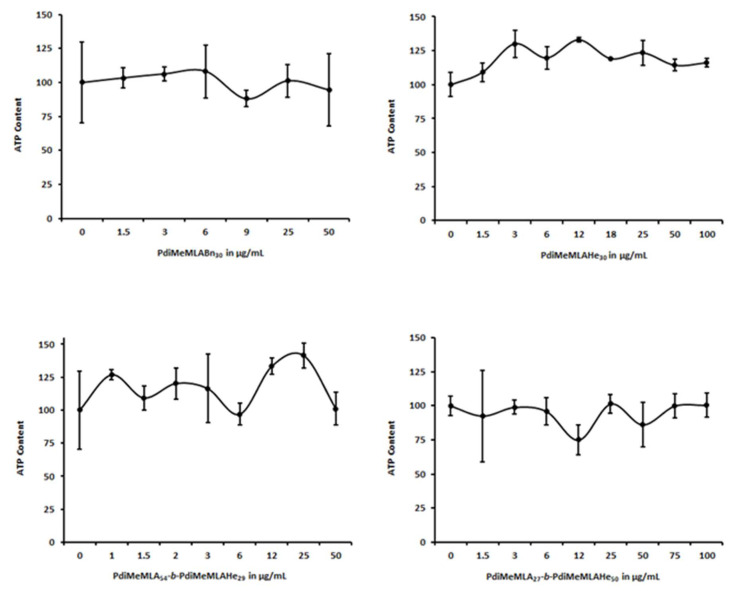
Differentiated HepaRG cell viability evaluated by measuring the ATP content at 24 h of incubation with increasing concentrations of (co)polymers PdiMeMLA_54_-*b*-PdiMeMLAHe_29_, PdiMeMLA_27_-*b*-PdiMeMLAHe_50_, PdiMeMLABn_30_ and PdiMeMLAHe_30_ NPs.

The progenitor (Figure 5) and the differentiated (Figure 6) cells have been exposed for 24 h to various concentrations of NPs prepared from different PdiMeMLA (co)polymers. Influences of the nature of lateral groups, namely benzyl and hexyl, of the hydrophobic homopolymers, and the hydrophilic/hydrophobic molar mass ratio of the block copolymer PdiMeMLA-*b*-PdiMeMLAHe on cell’s viability have been evaluated by measuring the intracellular content in ATP.

In progenitor HepaRG cells (Figure 5), the ATP content decreased in a dose-dependent manner for both the hydrophobic homopolymers and block copolymer PdiMeMLA-*b*-PdiMeMLAHe derived NPs. For the homopolymer-based NPs, the IC_50_ values (Table 7) revealed that PdiMeMLAHe_30_ based NPs are the least toxic one with an IC_50_ of 23.56 ± 2.12 µg/mL, while the IC_50_ of PdiMeMLABn_30_ based NPs was much lower at 6.95 ± 2.2 µg/mL. These data suggested that the nature of lateral groups of the hydrophobic homopolymers derivated from PdiMeMLA has a significant effect on the cytotoxicity of the corresponding NPs towards progenitor HepaRG cells. For PdiMeMLA_27_-*b*-PdiMeMLAHe_50_ NPs and PdiMeMLA_54_-*b*-PdiMeMLAHe_29_ NPs, the IC_50_ found were 22.11 ± 0.84 and 4.65 ± 0.49 µg/mL, respectively, demonstrating that the PdiMeMLA_54_-*b*-PdiMeMLAHe_29_-based NPs were much more toxic and that hydrophilic/hydrophobic molar mass ratio of block copolymer PdiMeMLA-*b*-PdiMeMLAHe strongly impacted the cell viability of progenitor HepaRG cells. The PdiMeMLA_27_-*b*-PdiMeMLAHe_50_ NPs were the least toxic since no significant decrease in the ATP content was observed up to 12 µg/mL.

Unexpectedly, the same concentrations of hydrophobic homopolymers and block copolymer PdiMeMLA-*b*-PdiMeMLAHe derived NPs showed no significant toxic effects towards differentiated HepaRG cells after 24 h of incubation up to 100 µg/mL of (co)polymers (Figure 6).

Such difference and behavior can be explained by the strong phenotypical changes between progenitor and differentiated HepaRG cells. In contrast to progenitor HepaRG cells that actively proliferate but show low expression levels of liver specific functions, differentiated hepatocyte-like HepaRG cells are quiescent and metabolically competent cells expressing most phase I, II, and III detoxifying enzymes [62,63,64]. It can be postulated that the detoxifying machinery of differentiated HepaRG cells catabolize and eliminate the polymers and organic compounds used during the synthesis and/or that the (co)polymers are more toxic towards proliferating cells.

**Figure 7:**
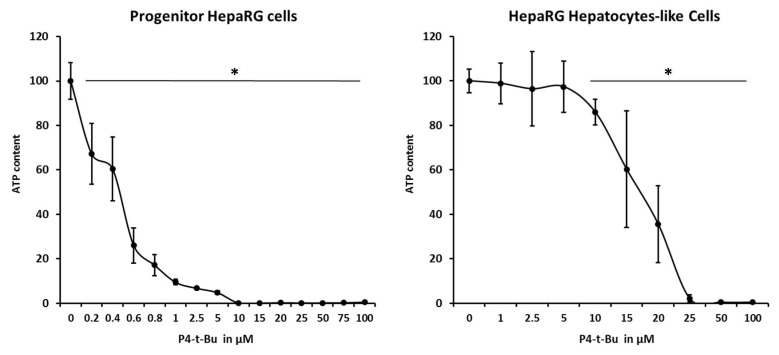
Progenitor and differentiated HepaRG cell viability evaluated by measuring the ATP content at 24 h of incubation with increasing concentrations of P_4_-*t*-Bu. Statistics: * *p* < 0.05 for treated cultures versus untreated control cultures (0).

Since ^1^H-NMR spectra of hydrophobic homopolymers and amphiphilic block copolymers have highlighted the presences of P_4_-*t*-Bu traces, we also evaluated the toxicity generated by this base in order to provide a possible explanation for the cytotoxicity observed with NPs especially in progenitor HepaRG cells. Both progenitor and differentiated HepaRG cells were thus incubated with increasing concentrations of the base, and cell viabilities were determined using the ATP test (Figure 7). P_4_-*t*-Bu triggered a strong cytotoxicity in progenitor HepaRG cells with an IC_50_ at 0.55 ± 0.1 µM while the toxicity of the base was much weaker in differentiated cells with an IC_50_ found at 16.65 ± 1.89 µM. These data suggest that the traces of P_4_-*t*-Bu detected in the polymeric materials may contribute to the observed toxicities in progenitor HepaRG cells.

**Figure 8:**
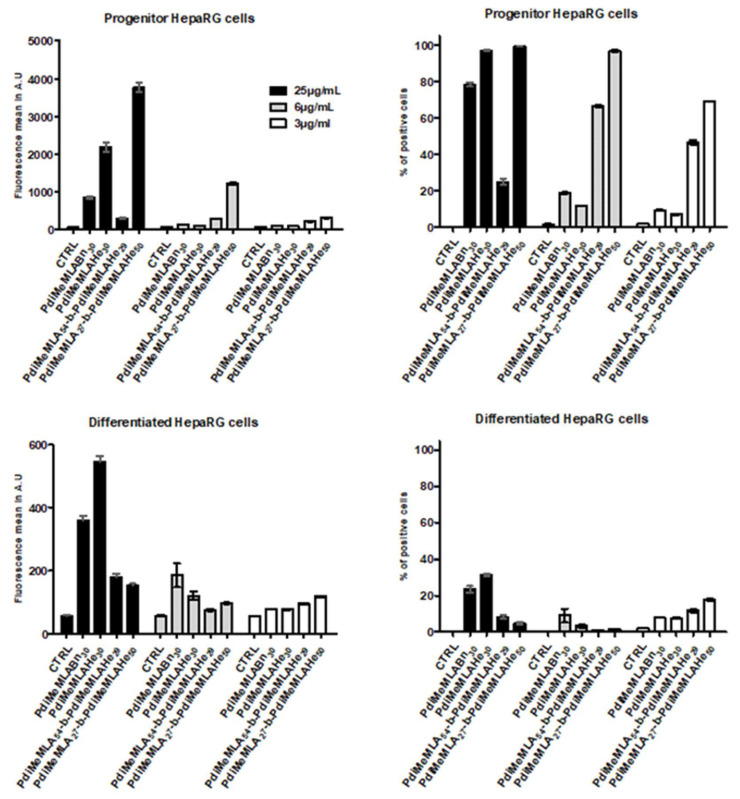
Percentages of positive cells and fluorescence intensities (arbitrary units, A.U.) measured for each DiR-loaded NPs in progenitor and differentiated HepaRG cells. NPs were prepared from the amphiphilic block copolymers PdiMeMLA_54_-*b*-PdiMeMLAHe_29_ and PdiMeMLA_27_-*b*-PdiMeMLAHe_50_, and the homopolymers PdiMeMLABn_30_ and PdiMeMLAHe_30_.

We next evaluated the cell uptake of homopolymer- and amphiphilic block copolymer-based NPs in both progenitor and differentiated HepaRG cells (Figure 8). Because of the significant cytotoxicity of some NPs towards progenitor HepaRG cells, we selected three concentrations (3, 6 and 25 μg/mL) of DiR-loaded NPs to assess their cell internalisation. After incubation of cells with NPs for 24, the viable cells were analysed by flow cytometry and two parameters were measured, the percentage of cells that had internalized fluorescent NPs (positive cells) and the mean of fluorescence reflecting the levels of NP accumulation in positive cells (Figure 8).

We found that the four NPs were much more internalized in progenitor cells than in differentiated cells since all cells were positive for PdiMeMLA_27_-*b*-PdiMeMLAHe_50_ NPs at polymer concentrations of 25 and 12 μg/mL and for PdiMeMLAHe_30_ NPs at 25 μg/mL. At the lowest concentration of 3 μg/mL, 70% of progenitor cells were positive for PdiMeMLA_27_-*b*-PdiMeMLAHe_50_ NPs. Interestingly, PdiMeMLA_54_-*b*-PdiMeMLAHe_29_ NPs that induced a strong cytotoxicity are relatively well internalized in progenitor cells at 3 and 12 μg/mL. The PdiMeMLABn_30_ and PdiMeMLAHe_30_ NPs showed much weaker uptake in progenitor cells with the exception of the PdiMeMLAHe_30_ NPs at 25 μg/mL, for which all cells were positive. As expected, the intensity of fluorescence correlated with the percentages of positive cells with the highest means of fluorescence found for the PdiMeMLA_54_-*b*-PdiMeMLAHe_29_ and PdiMeMLA_27_-*b*-PdiMeMLAHe_50_ NPs at the concentrations of 3 and 12 μg/mL and for PdiMeMLA_27_-*b*-PdiMeMLAHe_50_ and PdiMeMLAHe_30_ NPs at the highest polymer concentration of 25 μg/mL.

The higher cell uptake of PdiMeMLA_27_-*b*-PdiMeMLAHe_50_ NPs compared to that observed for PdiMeMLAHe_30_ NPs allows us to assume that the hydrophilic block eases the cell uptake of NPs whereas absence of any protective hydrophilic shell causes decrease in uptake. It is noteworthy that Huang et al. [65] have reported that NPs constituted by PMLABn allow a much higher uptake than NPs formulated from PEG-*b*-PMLABe. In the present study, the hydrophilic block composed by polymeric chains having carboxylic acid lateral groups confers a quite high negative surface charge (−30 mV to −42 mV) to the corresponding NPs compared to a quite neutral surface charge for PEG-*b*-PMLABe based NPs (−8 mV) [65]. The negative surface charge may allow more interaction with cell’s plasma membrane to explain the higher uptake of NPs prepared from such block copolymers.

In contrast, the NP’s internalization was less efficient in differentiated HepaRG cells since the highest percentages of positive cells were observed for PdiMeMLABn_30_ and PdiMeMLAHe_30_ NPs at the concentration in polymers of 25 μg/mL. It is noteworthy that the highest cell uptake in progenitor versus differentiated HepaRG cells were not obtained with the same NPs since PdiMeMLA_27_-*b*-PdiMeMLAHe_50_ NPs were well internalized in progenitor cells while cell uptake was more efficient for PdiMeMLAHe_30_ NPs in differentiated cells. In addition, the low cell uptake of all NPs by differentiated HepaRG cells also provided another explanation for the lower cytotoxicity observed in this cell model postulating that the decrease in cell viability is triggered by the accumulation of NPs within the cells.

Following the demonstration that DiR-loaded NPs were internalized with much higher efficiencies in progenitor HepaRG cells compared to their differentiated cell counterparts, we next compared the IC_50_ of the homopolymer- and amphiphilic block copolymer-based NPs in progenitor HepaRG cells, the estimated percentage of residual P_4_-t-Bu (% mol) associated with (co)polymers and the percentages of cells that internalized fluorescent NPs in cultures exposed with 6 and 25 μg/mL of (co)polymers to further analyze whether lower IC_50_ correlated with higher NP’s cell uptake and higher amounts of P_4_-t-Bu (Table 8). Although the amounts of the catalyst present in the batches of NPs are difficult to quantify precisely, we estimated that amounts of P_4_-t-Bu varied from 0.94 to 2.94% mol in the different batches of (co)polymers. Our data indicated that the IC_50_ did not correlate with the amounts of P_4_-t-Bu detected in the (co)polymers or the incubation with either the homopolymer- or amphiphilic block copolymer-based NPs. The toxicity was not related either to the level of NPs uptake, since we found similar levels of toxicity for PdiMeMLA_27_-b-PdiMeMLAHe_50_ and PdiMeMLAHe_30_ based NPs while the internalization levels were different, and a higher cytotoxicity of PdiMeMLAHe_30_ based NPs with lower NP internalization (Table 7).

This toxicity could also be linked to the physicochemical properties of NPs such as size, surface charge or shape. For example, the most toxic NPs PdiMeMLA_54_-*b*-PdiMeMLAHe_29_ has a size much smaller than other NPs (50 nm). Concerning the toxicity linked to the surface charge, it has been discovered that cationic NPs cause a more pronounced disturbance of the integrity of the plasma membrane, mitochondrial and lysosomal lesions, and induce the production of a higher number of autophagosomes than anionic NPs [66]. Additional studies will be necessary to further characterize the physicochemical parameters of our NPs.

## 4. Conclusions

In the present study, we report for the first time the synthesis of PdiMeMLA-based (co)polymers used to formulate NPs and to perform detailed analyses of their cell uptake and impact on cell viability in progenitor and differentiated HepaRG cells. We have observed cytotoxicity of NPs prepared from the hydrophobic homopolymers and amphiphilic block copolymer based NPs in HepaRG progenitor cells that may be triggered by the intracellular accumulation of NPs, the remaining traces of P_4_-*t*-Bu found in NPs batches and the low detoxification metabolism of these cells. Our results also suggest that the hydrophilic/hydrophobic ratio could play a role in the cytotoxicity observed with NPs formulated from the different PdiMeMLA-*b*-PdiMeMLAHe copolymers.

The studies of the NPs’ uptake by HepaRG cells have shown the significant differences in efficiency between homopolymers and amphiphilic block copolymer based NPs since the highest cell uptake in progenitor cells was obtained with the PdiMeMLA_27_-*b*-PdiMeMLAHe_50_ NPs while cell uptake was more efficient for PdiMeMLAHe_30_ NPs in differentiated cells, suggesting that the differentiation status of hepatic cells strongly modifies the NP–plasma membrane interaction and the cell process of NP internalization of PdiMeMLA (co)polymers-based NPs.

## Figures and Tables

**Table 1:** Characteristics of PdiMeMLABn_30_ and PdiMeMLAHe_30_.

Homopolymer	[Monomer]:[1-pyreneacetic acid]:[P_4_-*t*-Bu]	M_n, theo_ (g/mol)	M_n, NMR_^a^ (g/mol)	M_n, SEC_^b^ (g/mol)	Đ ^b^
PdiMeMLABn_30_	32:1:1	7500	7000	2700	1.20
PdiMeMLAHe_30_	33:1:1	7500	6800	5500	1.16

^a^ Determined by ^1^HNMR. ^b^ SEC analysis in THF/NEt_3_ (2w% of NEt_3_) at 35 °C.

**Table 2:** Characteristics of the two amphiphilic block copolymers prepared by sequential aROP of diMeMLAHe and diMeMLABn using 1-pyreneacetic acid/P_4_-*t*-Bu system.

Entry	[diMeMLAHe]_0_/[diMeMLABn]_0_ Initial Ratio	Theoretical Values		Experimental Values		M_n_^b^ (g/mol)	Đ ^b^
		M_PdiMeMLAHe_ (g/mol)	M_PdiMeMLABn_ (g/mol)	M_PdiMeMLAHe_^a^ (g/mol)	M_PdiMeMLABn_^a^ (g/mol)		
1	32:49	7300	11,500	6600	12,600	12,000	1.25
2	49:24	11,100	5700	11,800	6400	7400	1.31

^a^ Determined by ^1^H NMR.^b^ SEC analysis in THF/NEt_3_ (2w% of NEt_3_) at 35 °C.

**Table 3:** Characteristics of PdiMeMLA_54_-*b*-PdiMeMLAHe_29_ and PdiMeMLA_27_-*b*-PdiMeMLAHe_50_.

Block Copolymers	M_PdiMeMLA_^a^ (g/mol)	M_PdiMeMLAHe_^a^ (g/mol)	M_global_ (g/mol)	diMeMLA/ diMeMLAHe
PdiMeMLA_54_-*b*-PdiMeMLAHe_29_	7720 (n = 54)	6600 (m = 29)	14,320	50/50
PdiMeMLA_27_-*b*-PdiMeMLAHe_50_	3850 (n = 27)	11,800 (n = 50)	15,650	25/75

^a^ Determined by ^1^H NMR.

**Table 4:** Characteristics of NPs suspensions prepared from PdiMeMLABn_30_ and PdiMeMLAHe_30_.

Polymers	Dh (nm)	PDI	Zeta Potential (mV)
PdiMeMLABn_30_	100	0.19	−37
PdiMeMLAHe_30_	130	0.12	−53

**Table 5:** Characteristics of NPs suspension prepared by nanoprecipitation of amphiphilic block copolymers.

Copolymers	Dh (nm)	PDI	Zeta Potential (mV)
PdiMeMLA_54_-*b*-PdiMeMLAHe_29_	50	0.08	−36
PdiMeMLA_27_-*b*-PdiMeMLAHe_50_	150	0.17	−32

**Table 6:** Characteristics of DiR-loaded NPs suspensions.

Polymers	DiR (wt%)	Dh (nm)	PDI
PdiMeMLABn_30_	0.5	110	0.15
PdiMeMLAHe_30_	0.5	120	0.13
PdiMeMLA_54_-*b*-PdiMeMLAHe_29_	1	50	0.07
PdiMeMLA_27_-*b*-PdiMeMLAHe_50_	1	120	0.20

**Table 7:** Values of IC_50_ of the homopolymer- and amphiphilic block copolymer-based NPs in HepaRG cells.

NPs	PdiMeMLA_54_-*b*-PdiMeMLAHe_29_	PdiMeMLA_27_-*b*-PdiMeMLAHe_50_	PdiMeMLABn_30_	PdiMeMLAHe_30_
IC_50_ (μg/mL)	4.65 ± 0.49	22.11 ± 0.84	6.95 ± 2.2	23.56 ± 2.12

**Table 8:** Comparison of the IC_50_ of the homopolymer- and amphiphilic block copolymer-based NPs in progenitor HepaRG cells, the estimated percentage of P_4_-t-Bu (% mol) found in ^1^H-NMR spectra of (co)polymers and the percentages of cells that internalized fluorescent NPs (% of positive cells) quantified by flow cytometry in cultures exposed with 6 and 25 μg/mL of (co)polymers.

NPs		PdiMeMLA_54_-*b*-PdiMeMLAHe_29_	PdiMeMLA_27_-*b*-PdiMeMLAHe_50_	PdiMeMLABn_30_	PdiMeMLAHe_30_
IC_50_ (μg/mL)		4.65 ± 0.49	22.11 ± 0.84	6.95 ± 2.2	23.56 ± 2.12
P_4_-t-Bu (% mol)		1.3	0.94	2.94	2.87
Positive cells (%)	At 6 μg/mL	66.6	96.9	18.75	11.75
	At 25 μg/mL	24.8	99.5	78.4	97.3

## Supplementary Materials

The following are available online at https://www.mdpi.com/2073-4360/12/8/1705/s1, Figure S1: ^1^H NMR (500 MHz, Acetone-d6, 23 °C) spectrum of PdiMeMLABn.
