# Supplementary material for: Synthesis of Poly(Dimethylmalic Acid) Homo- and Copolymers to Produce Biodegradable Nanoparticles for Drug Delivery: Cell Uptake and Biocompatibility Evaluation in Human Heparg Hepatoma Cells
Source: Polymers (Basel). 2020 Jul 29;12(8):1705. doi: 10.3390/polym12081705 (PMC7464256; doi:10.3390/polym12081705)
Supplement: Supplementary file 1 [file polymers-12-01705-s001.pdf]

Supplementary Information

Synthesis of poly(dimethylmalic acid) homo- and copolymers to produce biodegradable nanoparticles for drug delivery: Cell uptake and biocompatibility evaluation in human HepaRG hepatoma cells.

Ali Khalil[1,2†$], Saad Saba[3†], Catherine Ribault[3], Manuel Vlach[3], Pascal Loyer[3*], Olivier Coulembier[1,*], Sandrine Cammas-Marion[2,3*]

[1] Laboratory of Polymeric and Composite Materials, Center of Innovation and Research in Materials and Polymers (CIRMAP), University of Mons (UMons), Place du Parc 23, 7000 Mons, Belgium.
[2] Univ Rennes, ENSCR, CNRS, ISCR (Institut des Sciences Chimiques de Rennes) - UMR 6226, 35000 Rennes, France.
[3] INSERM, INRAE, Univ Rennes, Institut NUMECAN (Nutrition Metabolisms and Cancer) UMR_A 1341, UMR_S 1241, F-35000 Rennes, France.

* Correspondence: *Sandrine.marion.1@ensc-rennes.fr* (S.C.M.); *olivier.coulembier@umons.ac.be (O.C.); pascal.loyer@univ-rennes1.fr (P.L.)*

† *Ali Khalil and Saad Saba have contributed to this work equally.*
$ *Current address:* LCPM, UMR CNRS/Université de Lorraine 7375, BP 20451, 54000 Nancy, France

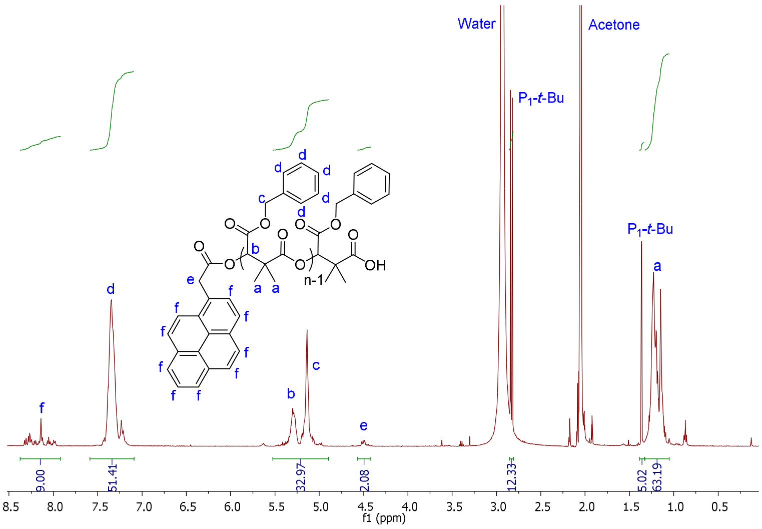

***Figure S1.** ^{1}H NMR (500 MHz, Acetone-d6, 23 °C) spectrum of PdiMeMLABn.*
